# Understanding engagement with digital health interventions designed for adults with hearing loss and tinnitus: a mixed-method systematic review

**DOI:** 10.1093/tbm/ibaf028

**Published:** 2025-06-30

**Authors:** Akshaya Ravichandran, Melanie A Ferguson, Wilhelmina H A M Mulders, Robyn S M Choi, Rebecca J Bennett

**Affiliations:** School of Human Sciences, University of Western Australia. 35 Stirling Hwy, Crawley, WA 6009, Australia; Ear Science Institute Australia, 2/1 Salvado Rd, Subiaco, WA 6008, Australia; School of Human Sciences, University of Western Australia. 35 Stirling Hwy, Crawley, WA 6009, Australia; Curtin School of Allied Health, Faculty of Health Sciences, Curtin University, Kent Street, Bentley Western Australia 6102, Australia; Curtin enAble Institute, Curtin University, Kent Street, Bentley, WA 6102, Australia; School of Human Sciences, University of Western Australia. 35 Stirling Hwy, Crawley, WA 6009, Australia; School of Human Sciences, University of Western Australia. 35 Stirling Hwy, Crawley, WA 6009, Australia; School of Human Sciences, University of Western Australia. 35 Stirling Hwy, Crawley, WA 6009, Australia; Ear Science Institute Australia, 2/1 Salvado Rd, Subiaco, WA 6008, Australia; Curtin enAble Institute, Curtin University, Kent Street, Bentley, WA 6102, Australia; National Acoustic Laboratories, University Ave, Macquarie Park, NSW 2109, Australia

**Keywords:** teleaudiology, e-health, m-health, engagement, Digital Health Interventions

## Abstract

**Background:**

Hearing loss and tinnitus are pervasive disabilities globally, which significantly impact individuals’ quality of life. Integrating Digital Health Interventions (DHIs) with traditional audiological management has proven beneficial for hearing loss and tinnitus management. Although it is established that DHI engagement is important for the real-world effectiveness of DHIs, there is a lack of systematic evidence aiming to understand engagement with DHIs in audiology.

**Purpose:**

This systematic review identified factors associated with hearing healthcare DHI engagement to inform future DHI development and research in audiology.

**Methods:**

Adhering to Synthesis without Meta-Analysis guidelines, we conducted a mixed-methods systematic review using a convergent integrated approach. A comprehensive search across seven databases until December 16, 2023, identified 62 studies meeting inclusion criteria. Data extraction involved modifying the Joanna Briggs Institute (JBI) extraction form and deductive coding using the Perski et al. (2017) framework to identify factors related to engagement.

**Results:**

The review revealed a diverse range of factors associated with DHI engagement in the audiology literature.

**Conclusion:**

Analysis within the Perski et al. (2017) framework highlighted the importance of user-related constructs, such as enhancing DHI accessibility, empowering users, and aligning DHIs with user needs and lifestyles in facilitating engagement. Due to the limited number of studies focusing on engagement as the primary outcome, we based our inferences on secondary outcomes and discussions from the available literature. While this review consolidates existing knowledge on engagement, it underscored the imperative for more in-depth investigations into engagement with hearing healthcare DHIs.

**Implication statement**

**Practice:**

Hearing healthcare clinicians should consider their clients’ lifestyles and daily routines prior to recommending DHIs to minimise disruptions to everyday life and enhance DHI engagement in real-world settings.

**Policy:**

Policy can help promote seamless integration of DHIs into users’ daily routines by incentivising the adoption of person-centred approach in DHI development and research; this may help reduce disruptions, ensure that DHIs are tailored to meet users’ specific needs and preferences, and enhance real-world effectiveness of DHIs.

**Research:**

There is limited in-depth exploration of how adults with hearing loss and/or tinnitus engage with DHIs in audiology, and future research should explore engagement with DHIs as a primary concept and could look into some of the areas suggested in the review such as the link between empowerment and engagement.

## Introduction

Hearing loss and tinnitus (the perception of phantom sounds, often described as ringing in the ears) often coexist in individuals [1, 2], and are prevalent chronic health conditions worldwide [3, 4]. In 2019, it was estimated that 1.5 billion people in the world lived with hearing loss, of whom, 430 million found it disabling [3]. Tinnitus affects 14% of the world’s population, and 2% of the world’s population finds it disabling [4]. The impacts of hearing loss and tinnitus on an individual are multifactorial and the conditions can significantly impact an individual’s quality of life [5, 6]. Hearing loss can negatively affect an individual’s ability to communicate, resulting in increased participation restrictions, social isolation, relationship difficulties, and reduced physical and mental well-being [7–10]. This, in turn, can lead to feelings of anxiety, depression, and loneliness [11–14] Tinnitus can cause sleep and concentration difficulties, resulting in indirect psychosocial impacts such as hopelessness, anxiety, and depression [4, 15, 16].

Given the complex and generally chronic nature of hearing loss and tinnitus, comprehensive audiological rehabilitation is required to self-manage these conditions. Comprehensive audiological rehabilitation is informed by four cornerstones which include, (i) sensory management (e.g. provision of hearing aids and other assistive devices), (ii) education (improving hearing-related knowledge and skill), (iii) auditory cognitive training (perceptual training), and (iv) motivational engagement (collaborative decision making and counselling) [17, 18]. One review examined how recent studies in audiology have expanded on the four cornerstones, highlighting a growing emphasises on the electronic and mobile health delivery of aural rehabilitation [17]. These emerging models of health service delivery are thought to improve personalisation and interactivity, leading to individuals who are better informed and empowered to self-manage their hearing loss [17, 19].

Electronic and mobile-delivered health interventions can be referred to as digital health interventions (DHIs) [20]. DHIs leverage digital technologies, such as mobile applications, smartphones, and the internet to deliver health-related services, support, and information [20, 21]. The World Health Organization classifies DHIs into four main categories: client intervention, healthcare provider interventions, health system or resource managers interventions, and interventions for data services [22]. This review focuses specifically on client interventions, i.e. DHIs targeting individuals managing their own healthcare [22]. Client interventions can be self-guided or involve healthcare provider support; this study specifically examined self-guided DHIs. In audiology, self-guided DHIs serve various functions, including hearing loss screening, hearing aid self-fitting and tuning, auditory training, tinnitus management, and sound therapies for tinnitus, providing cost-effective, safe, and scalable audiological support [19, 23–28].

### Engagement with digital health interventions

Ensuring meaningful engagement with DHIs is vital to ensure individuals adopt DHIs and find them effective in real-world scenarios [29–32]. Engagement can be described as a complex, multi-dimensional interaction between the user and intervention [30, 32–34], and is attributed to three main dimensions: behavioural, cognitive, and affective [33]. Behavioural engagement is the user’s involvement in, utilization of, and adherence to the DHI [30, 33, 34]. Cognitive engagement refers to the extent to which users agree with the DHI’s rationale and perceive the DHI as suitable to their goals [34]. Affective engagement describes the user’s subjective experience with the intervention, which is characterized by their emotional response, attention, and interest in the DHI [30, 33, 34].

### Rationale for systematic review

Although some studies evaluate engagement with DHIs in audiology [35–37], their scope of what constitutes engagement and what factors influence engagement differ. For example, one study [38] evaluated engagement with a DHI called m2hear using the User version of the Mobile Application (uMARS) Scale [39] that attributes engagement with a DHI to four key dimensions, entertainment, user interest, interactivity, and personal relevance. Improvements in these dimensions are assumed to facilitate DHI engagement [39]. Another study [37] extended the understanding of engagement in audiology by investigating users’ subjective experiences in interacting with a DHI through semi-structured interviews. This in-depth investigation identified that factors such as motivation to change health-related attitudes, user expectations, personal relevance, and the DHI’s overall appeal influenced user engagement with DHIs. The two studies emphasize different aspects of engagement. One emphasized quantifiable dimensions like interactivity and personal relevance, while the other emphasized users’ subjective experiences and motivational factors. This indicates a need to synthesize diverse findings and build a comprehensive evidence base that holistically reflects our current understanding of engagement in audiology, and the factors associated with it.

To date, no systematic review has specifically evaluated the evidence concerning the factors associated with DHI engagement in audiology. Previous systematic reviews on DHIs in audiology have identified and classified mobile applications available for hearing health care [27, 28], examined the role of DHIs in the patient journey of adults with hearing loss [40], and assessed the effectiveness of DHIs [41, 42]. However, discussions of these reviews are largely centred on features of intervention (i.e. the content and delivery of the intervention), with limited exploration of how user-related constructs and broader environmental factors can influence DHI effectiveness and uptake. While understanding the effective features of a DHI is crucial for future hearing healthcare DHI development, the next step is to complement these findings by systematically reviewing factors associated with DHI engagement, which includes user-related and broader environmental constructs. This is important because a high-quality, impartial body of evidence is necessary to understand how engagement can potentially be improved in hearing-related DHIs. The findings can help inform future research, guide the development of engaging and effective DHIs, and help hearing healthcare clinicians promote better client engagement with DHIs. The objective of this systematic review was to identify factors that influence engagement with DHIs designed for adults with hearing loss and/or tinnitus. The research questions this review aimed to address were

(1) What are the factors associated with hearing healthcare DHI engagement?
(2) What are the implications of the factors for future DHI development and research in audiology, as informed by broader literature and discussions?

## Methods

This review followed the methodology guidelines developed by the Joanna Briggs Institute (JBI) for mixed-methods systematic review (MMSR) [43]. An MMSR combines findings from qualitative, quantitative, and mixed-methods primary studies [43]. By including diverse forms of evidence from diverse types of research, MMSRs can maximise findings to generate robust evidence [43].

JBI recommends following the convergent integrated approach if the MMSR research question can be addressed by both quantitative and qualitative studies [44]. We aimed to gather evidence on engagement, whether reported as a primary or secondary outcome, or discussed peripherally in studies primarily focused on the effectiveness or uptake of DHIs. This approach enabled us to comprehensively explore all evidence related to factors associated with engagement, in line with the methodologies used in previous reviews [45, 46]. Given that outcomes and discussions of both qualitative studies (e.g. qualitative interviews exploring user experiences with DHIs) and quantitative studies (e.g. randomized controlled trials assessing the effectiveness of DHIs) can explore factors linked to engagement, the convergent-integrated approach combining extracted data into a single-pooled synthesis was selected.

Data transformation is an integral part of the convergent integrated approach, which involves transforming extracted data into a consistent format, (i.e. either qualitative or quantitative) [44]. In this review, relevant quantitative data was transformed into qualitative data as this is considered less error-prone than assigning numerical values to qualitative data. This entailed rewriting relevant quantitative extracts (e.g. statistical findings) into textual descriptions. Given that most of the relevant data on engagement were already present as textual descriptions (i.e. already qualitized), only relevant extracts from n=6 studies were qualitized. An example of qualitized data is provided below:

### Original data from included study

“Results showed that all subjects were highly autonomously motivated (average: 5.9, SD = 0.8, range: 3.5–7.0) with a variable amount of controlled regulation (average: 2.8, SD = 1.6, range: 1.0–6.6)” [47].

### Qualitised data

“Subjects were highly autonomously motivated to engage the DHI in the study. It is difficult to determine the extent to which intrinsic desire and external encouragement played a role in their decision”

Please note that with qualitized data, the transformation process results in a qualitative synthesis and analysis of the data, with no statistical methods required, as outlined by JBI guidelines [43, 44]. The protocol for the review was registered with the International Prospective Register of Systematic Reviews (PROSPERO) ID: [blinded for review]. The review follows Synthesis Without Meta-Analysis (SWIM)-guidelines (supplementary material 1).

### Conceptual framework

Given that data on engagement were extracted from studies that do not primarily explore engagement, the Perski et al. (2017) conceptual framework on factors influencing engagement with behaviour change DHIs was used to guide data extraction to ensure the validity of extracted data. While the framework was originally developed for behaviour change DHIs, we have employed it to guide data extraction in this review for the following reasons.

- Unlike previous frameworks confined to a single scientific field, the framework draws on theoretical predictions and empirical observations across multiple interrelated fields.
- The framework is evidence-based, specific to behavioural change DHIs, and currently provides the most comprehensive list of factors influencing engagement. A significant proportion of DHIs in audiology aim to enhance self-management of hearing loss and tinnitus, inherently classified as behavioural change DHIs. Thus, this framework is well-suited to our analytical approach.

Factors influencing engagement are organized under five overarching concepts discussed in the Perski et al. (2017) framework; these include context, delivery, mechanisms of action, population, and setting.

**Figure 1:**
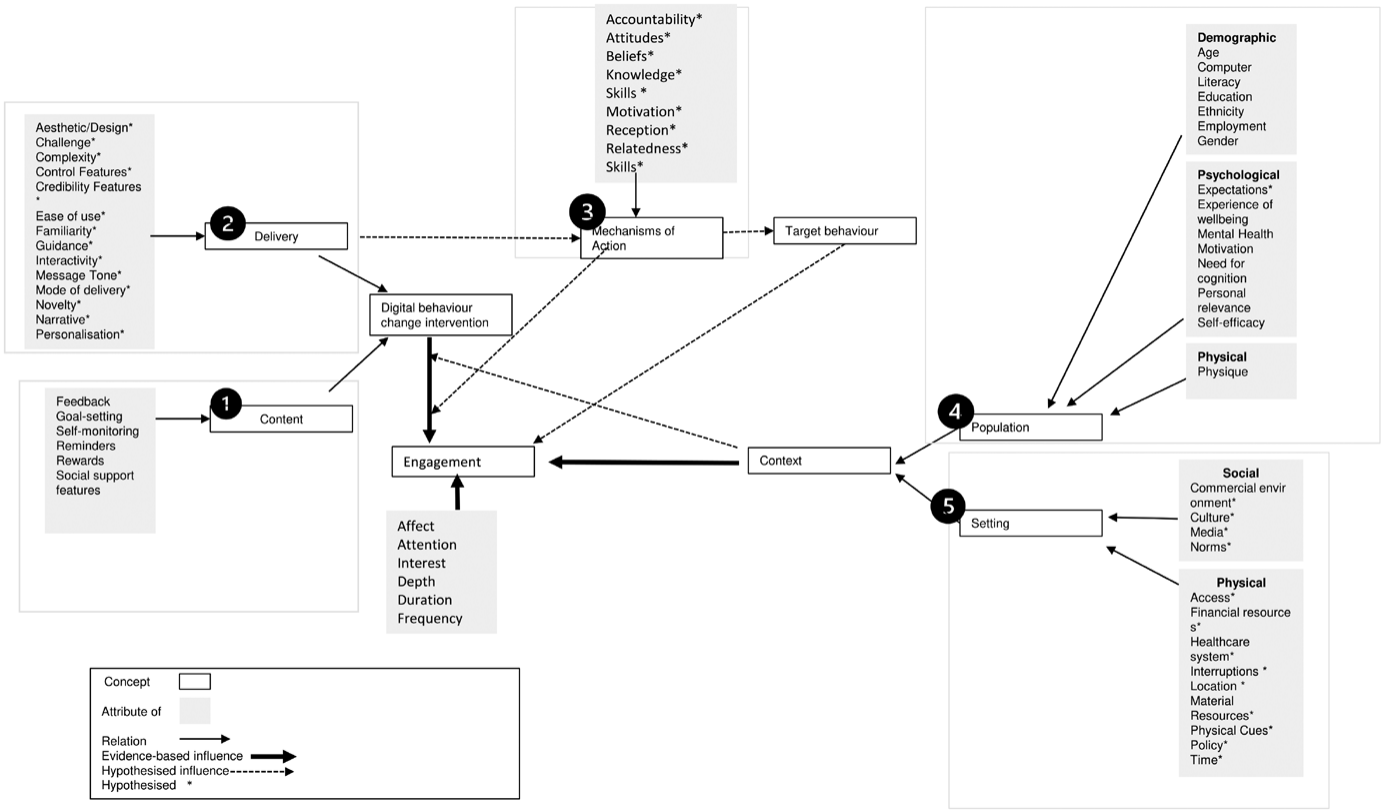
*Perski* et al. *(2017) Framework on Factors influencing engagement (adapted).* Note: Adapted from From Perski, O., Blandford, A., West, R., & Michie, S. (2017). “Conceptualising engagement with digital behaviour change interventions: a systematic review using principles from critical interpretive synthesis.” Translational behavioral medicine, 7(2), 254-267. Copyright. Distributed under Creative Commons 4.0 licence. https://creativecommons.org/licenses/by/4.0/ The attributes of the five highlighted concepts (content, delivery, mechanisms of action, population, and setting) are considered factors influencing engagement with DHI in this study. The attributes of engagement (i.e. affect, attention, interest, depth, duration, and frequency) are dimensions of engagement. That is, Perski et al. (2017) define engagement as the extent of use, characterised by depth, duration, frequency and the subjective experience, characterised by affect, attention and interest. Attributes of engagement are not considered factors influencing engagement as they are dimensions that constitute engagement.

- Content highlights *what* content is presented to users, and includes factors such as feedback, and goal setting.
- Delivery highlights *how* the content of the DHI is implemented and includes factors such as complexity and ease-of-use.
- Mechanisms of action describe how the DHI is expected to lead to its effects and include factors such as change in user’s knowledge and motivation.
- Population describes factors related to individuals who use the DHI and includes factors such as age and physique.
- Setting explains the broader setting in which the DHI is used and includes factors such as culture and healthcare systems (Figure 1).

### Search strategy

Seven allied healthcare and psychological databases were comprehensively searched: APA PsycINFO, CINAHL, Embase, Global Health, Cochrane Central, MedLine and PubMed. Search strategies were developed for each database using Boolean operators, truncation, and Medical Subject Headings (MeSH), adapting to the database’s syntax (see Supplementary Material S2). AR performed an initial literature search and consulted a librarian in modifying and further developing the search strategies. The search was performed from the earliest possible date up to and including the 16 December 2023 and only articles in English were included. Additional studies were identified manually through snowballing of the reference lists from included studies.

### Selection criteria

The selection criteria follow the PICo criteria for qualitative systematic reviews, where P is the population of interest, I is the phenomena of Interest, and Co is the Context [48].

#### Study design

Primary qualitative, quantitative, and mixed-method studies were included. Grey literature, non-peer-reviewed literature, expert opinion, reviews, protocol, practice guidelines, editorials, letters, book chapters, conference abstracts, and other studies that do not report the results of the original, empirical investigation were excluded.

#### Population

The following populations were included:

- Adults with hearing loss and/or tinnitus as defined by the study.
- Studies where participants were both adults with hearing loss and/ or tinnitus, and adults with normal hearing were included if the majority of participants were adults with hearing loss and/or tinnitus as defined by the study.

We excluded populations of children (<18 years of age), and adults with normal hearing as defined by the study. Please note, ‘normal hearing’ is the term used across the broader literature, and we recognise that definitions of normal hearing can vary across studies. For example, some studies defined normal hearing based on self-reported measures, while others used audiometric classifications. As such, we accepted any clear definition of ‘normal hearing’ outlined by the study.

#### Phenomenon of interest

To be included DHIs had to be self-guided, delivered via the internet or through mobile technologies, and must target hearing loss and/or tinnitus. DHIs were excluded if they were designed for children. DHIs wherein healthcare clinicians play a primary role in guiding the care of adults with hearing loss and/or tinnitus.

#### Context

Studies conducted in any country or setting were included.

### Study selection

Search results were uploaded to Covidence, a web-based systematic review management tool. Duplicates were removed via Covidence prior to the screening of titles and abstracts. AR and a research assistant screened titles and abstracts against the inclusion and exclusion criteria. Supplementary material 3 shows the results of the initial searches, screening, and selection processes and is reported according to PRISMA guidelines [49]. Of the 1376 abstracts screened, discrepancies arose in 20 cases, which were discussed and resolved with RB. Three abstracts could not be resolved at this stage, so they were included in the 166 full texts reviewed. Full texts were reviewed by AR and a research assistant; discrepancies arose in 10 out of 166 full texts, which were discussed with RB, and consensus was reached on a decision.

### Data extraction

AR and a research assistant independently extracted the data, adopting a predefined matrix, which was developed by modifying fields in the JBI data extraction form [50]. AR modified the form and piloted data extraction on two included studies. RB and MF further refined the form to include the following fields: first author, year of publication, name of DHI, description of DHI, implementation, study type, participant characteristics, setting, delivery factors, content factors, mechanisms of action factors, population factors, and setting factors.

To categorize the data into the different factors relating to engagement, AR and a research assistant independently and deductively coded all methods, results, and discussion sections of included studies, employing the Perski et al. (2017) framework. Discrepancies in coding were discussed between AR and the research assistant and an agreement was reached. In cases where coders were unsure of the categorization, text was highlighted but not categorized, and flagged for discussion, and a decision was made following discussion. Once coding was completed, RB reviewed the codes and their corresponding excerpts to further ensure validity. Extracted data are presented in Supplementary Material 4.

### Assessment of methodological quality

All included papers were critically appraised by two independent reviewers (AR and a research assistant) using appropriate JBI checklists [50]. Studies were critically appraised using Yes, No, Unclear, and Not Applicable responses to the checklists. Details of the appraisal are reported in Supplementary Material 5. A narrative description is also presented in the results.

## Results

### Study characteristics

Of the 62 studies included in this systematic review, 44 were quantitative, 11 were qualitative, and seven were mixed-method studies. Of the 44 quantitative studies, 12 were randomized controlled trials (RCTs) examining the effectiveness of DHIs, and 32 were cross-sectional studies validating the accuracy and reliability of DHIs. The 11 qualitative studies were all phenomenological studies incorporating a range of methods such as focus groups, think-aloud interviews, and semi-structured interviews to investigate user experiences with DHIs. The seven mixed-method studies incorporated qualitative methods such as semi-structured interviews and focus groups with quantitative surveys and questionnaires.

Regarding study setting, 29 studies were conducted in research settings, five in hospital/ clinical settings, 18 in participants’ homes/online, and two were conducted in wider community settings. In 11 studies, DHIs were provided in a research setting, and participants used them in everyday life. With regards to DHI implementation, 24 studies provided participants with a loan device, and 26 studies had participants access the DHI on their personal devices. In one study, participants could opt to access the DHI on their personal devices or obtain a loan device. In the remaining 12 studies, it was either unclear how the DHI was accessed, or participants did not use a DHI and instead discussed the conception of a DHI.

Among studies that discussed DHIs for hearing loss, 12 discussed self-performed hearing tests, ten discussed educational and informational tools, and nine discussed hearing aid self-fitting and/or fine-tuning apps. Five studies explored DHIs that facilitate communication between the Deaf and healthcare clinicians. Three studies investigated auditory training DHIs and two discussed sound-amplification apps. One study discussed the conception of a virtual hearing clinic, which included both hearing tests and hearing aid self-tuning. Another study discussed a decision aid for audiology care, and one study discussed a self-selection of frequency allocation tables for CI users.

Regarding tinnitus, eight studies discussed DHIs designed for the psychosocial management of tinnitus. Four discussed sound therapy for tinnitus, two discussed a DHI that tracks tinnitus severity, one discussed tinnitus pitch matching and one study discussed an educational DHI for tinnitus. One study broadly discussed tinnitus apps.

### Quality appraisal

The methodological quality varied across different studies. For the 39 cross-analytical and mixed-methods studies, data collection, recruitment, and sample characteristics were appropriate. 12 did not clearly identify confounding factors and 18 did not clearly state strategies to deal with confounding factors. Six studies did not clearly state the inclusion criteria for the sample and 11 studies did not clearly describe the settings and subjects. Two studies received no or unclear responses for using appropriate statistical methods.

Outcomes for all 12 RCTs were measured reliably and in the same way for treatment groups. Ten RCTs received either no or unclear responses for blinding outcome assessors to control and treatment groups. Nine RCTs received either no or unclear responses for blinding treatment assessors to treatment assignment. In two RCTs, allocation to treatment groups was not concealed and it was unclear whether two RCTs achieved true randomisation.

All 11 qualitative studies had congruity between the research question, methodology, methods used to collect data and data analysis. Two qualitative studies were missing statements on reflexivity that discussed the impact of the researcher on the research and five studies were missing a statement locating the research culturally or theoretically.

### Engagement factors across included studies

Forty seven of the 62 studies included in this review discussed factors associated with engagement. We included all research on self-guided DHIs in audiology that met our inclusion criteria to ensure that no factors related to engagement with self-guided DHI were overlooked. Following the methodology outlined by JBI and as detailed in our PROSPERO submission, we were only able to identify which of the included studies discussed engagement after double-blinded extraction of the data. Although we had a separate category called “other” to capture factors that did not fall under the Perski et al. (2017) framework, we did not identify factors that were not discussed in the framework; thus, all factors and concepts discussed in this section are derived from the framework.

This section provides an overview of factors associated with engagement identified in the reviewed studies. It is organized into five subsections, each focusing on one of the five concepts associated with DHI engagement [30]. Each subsection outlines the different factors within the concept, notes the number of studies reporting each factor, and summarises how these factors manifest in the audiology literature with illustrative quotes. To further contextualize factors linked to engagement within the audiology literature, the table distinguishes the factors as barriers and facilitators.

#### Content

*N* = 13 studies reported on factors linked to content (i.e. what features are presented to users within a DHI). We identified factors linked to content, which included feedback, self-monitoring, social support features, reminders, and rewards (Table 1).

**Table 1.** Factors that fall under the ‘content’ concept

Factors within the concept	Number of studies reporting factor	Barrier/Facilitator/Mixed	Summary of main findings for each factor as it applies to the audiology literature	Illustrative quotes from included studies
Feedback	7	Facilitator	Feedback offers an opportunity to reinforce newly acquired knowledge and self-monitor progress, enhancing engagement with the content provided by the DHI [47, 51]Tailored feedback ensures that the feedback aligns with the user’s specific needs, enhancing engagement with the DHIs content [47, 51–54, 66,89]	“Visual feedback was provided as to whether the response was correct or incorrect, and the correct answer was also shown if an incorrect answer was typed, because the purpose of the identiﬁcation level is to train the participant to identify which sound was perceived” [89]
Self-monitoring	5	Facilitator	The ability to track and reflect on one’s progress provided an avenue for active involvement and engagement with the DHI [47, 51, 52, 66, 55]	“Users saw potential benefits of using a self-monitoring tool such as the THI, allowing them to gain feedback on their progress toward their tinnitus-related goals as well as a sense of achievement and confidence that the intervention was beneficial” [66].
Social support features	4	Facilitator	Features that allowed users to connect with others (e.g. discussion forums and testimonials from individuals), improved the perceived credibility of the DHI providing an avenue for engagement with the DHI’s content [52, 56, 57, 88]	“The study concluded that PHIs perceived advantages of the online program…. They appreciated being empowered on learnings about [hearing impairment], and they especially appreciated testimonial videos from peers” [52].
Reminders	1	Facilitator	Users demonstrated engagement with reminders when they had control over the way in which they received them [86]	“Other than that, in-app notifications, which are alerts sent from the app to the mobile device to remind them when to collect their medication, were preferred compared to conventional text messages, as they were perceived to be more convenient” [86]
Rewards	1	Facilitator	Rewards were strategically offered at different stages of the training process to motivate users and encourage sustained engagement with a training program [47]	“During the rehabilitation process, the user collects ‘badges’ to represent milestones in performance as they are reached……... In order not to discourage the participants, they were not informed regarding the total number of badges that can be earned. In our study, each participant could earn the same six milestones. These milestones were not particularly ambitious (e.g. use your sound processor for 25 hr) but were set up to motivate the participants to keep using the MHA during the 4-week study period. Previous research has taken into account gamification for seniors in order to increase adherence to eHealth applications” [47]

#### Delivery

*N*= 45 studies in this review reported on factors related to the delivery concept (i.e. how the content is presented within the DHI). Under this concept, we identified factors such as personalisation, ease of use, complexity, guidance, mode of delivery, interactivity, aesthetic/design, challenge, message tone, novelty, and narrative (Table 2).

**Table 2.** Factors that fall under the ‘delivery’ concept

Factors within the concept	Number of studies reporting factor	Barrier/Facilitator/Mixed	Summary of main findings for each factor as it applies to the audiology literature	Illustrative quotes from included studies
Personalisation	17	Facilitator	Customising content to meet individual needs enhanced the accessibility of personally relevant information, facilitating engagement with the DHI [38, 47, 51, 52–54, 55, 56, 57, 58–63, 64, 65]	“From the patient’s perspective, participants were very keen on having access to the Recipient Portal. Research has shown that having access to personalized information is more useful compared with having access to general information of patients with chronic conditions... In the current study, most participants found it very useful having personalized information (e.g. warranty information) at a single location, accessible at any time” [47]
Ease of use	18	Facilitator	Clear instructions, concise information, simplified tasks, and a user-friendly interface promoted engagement with the DHI [37, 38, 51, 52, 66, 56, 58, 62, 67–70, 71–76]	“It wasn’t loads of diving off into other areas and that, it was…nice and simple. There wasn’t anything complicated” (M067). It was also well-organized and structured, ensuring that information could be easily located: M062 said, “It’s very easy to get to the section you need to look at or you want to look at…you can see quite clearly which elements you need to go to” [76].
Complexity	20	Barrier	Complex and non-intuitive navigation within DHIs acted as barriers to engagement [56]; conversely, engagement with the DHI was facilitated by employing simple, easy-to-understand language, and straightforward navigation [37, 52, 54, 55, 66, 57, 58, 59, 70–72,77, 64, 76,78–82]	“We needed log-ins and user names to answer the weekly assignments, and that wasn’t clear enough for me. [Those responsible for the program] made all the technicalities too complicated/…/it all made me miss out on participating” [56].
Guidance	13	Facilitator	In the absence of a clinician to guide participants, instructions were provided to assist in engaging with tasks such as performing pure-tone audiometry, guided relaxation and programming hearing aids [37, 52, 66, 58, 68–70, 74, 79, 64,83–85]	“When developing the WordRec app, we had the goal of making the test instructions clear enough that patients could easily take the test without any help from a professional. We found that about half of the subjects we tested required some help with the app, mostly with basic iPhone usability issues such as placing the EarPods and adjusting the volume. We hope to make the instructions clearer by improving the user interface; however we expect that there will still be many patients with hearing loss who are not familiar enough with a smartphone to take the test without assistance [68]”
Mode of delivery	9	Mixed	Adults with hearing difficulties generally conveyed a preference for accessing health interventions through their mobile devices over alternatives such as booklets, DVD-delivered interventions, or computer-based interventions [56, 86, 58, 73, 76, 80–82, 87]. This inclination was attributed to the portability of mobile devices and the widespread use of such devices compared to other available options.	“It’s more convenient to use, wherever you are. You can just get your phone out, whereas you might not have the booklet with you” [76].
Interactivity	10	Facilitator	Interactive features such as discussion forums and quizzes promoted active participation in the content delivered by the DHI, facilitating engagement [51, 56–58, 86,88, 75, 76, 81, 64]	“Both individualized and interactive elements were incorporated into the design of the mobile platform for delivery of the intervention, a process that was iterative and followed a user-centered and participatory design approach. Therefore, although some participants expressed ambivalence, most stated that they used m2Hear because they believed that both the mRLOs and interactive components would improve their knowledge of hearing aids and communication, resulting in more successful outcomes” [76]
Aesthetic/Design	5	Mixed	Design features that simplified the intervention for users, such as audio-visual formats [86, 69], readable fonts and colours [58], and gamification [84], facilitated engagement. Conversely, unwelcoming and unprofessional designs acted as barriers to engagement [46].	“Besides providing text descriptions regarding medication queries, participants felt providing video directions was necessary as not all Deaf individuals were literate. Similarly, participants noted a preference for visuals in video form accompanied by subtitles, with a suggestion for captions in multiple languages” [86].
Challenge	5	Facilitator	Quizzes and the automated re-presentation of incorrect responses in training activities played a constructive role in reinforcing the knowledge and skills imparted by the DHI, facilitating engagement with the DHI’s content [35, 89, 55, 88, 76]	“In addition, the quizzes and interactive features also ensured that the content of the mRLOs [mini Reusable Learning Objects] were successfully retained and remembered, “Yes, it was like a little test to work on my memory. Even though I was watching the [mRLO] I was probably not watching it, whereas a quiz it was making me remember stuff” [76]
Message Tone	3	Mixed	Conveying health information in a positive and respectful manner facilitated engagement with the DHI [37, 87]. Contrastingly, disrespectful and condescending content about one’s hearing loss, acted as a barrier to engagement [57].	“In addition, they [participants] disliked the confrontational aspect of the story (the family depicted in the animation told the character with HL that they would stop visiting him frequently until he sought help). Therefore, we cancelled our plans for using the animated storyline” [57]
Novelty	2	Barrier	When DHIs failed to provide new or substantial content to users, the DHI was perceived as pointless, leading to user disengagement [47, 66]	“[A] participant commented that she felt she was already sufficiently proficient at managing her CI and that this tool [the DHI] did not provide her with additional useful information” [47].
Narrative	1	Facilitator	Presenting health information through a “drama” format helped simplify complex health content, promoting easy comprehension and facilitating user engagement with the content of the DHI [80]	“When there is drama, you get to understand something you never understood, so it’s very good. In addition, a few Deaf participants wished to review the dramas at their own time and place of convenience” [80]

#### Mechanisms of action

*N* = 13 studies reported on factors linked to mechanisms of action (i.e. how the DHI achieves its desired outcomes). Factors like change in users’ knowledge, skill, motivation, attitudes and beliefs after using a DHI were identified under this concept (Table 3).

**Table 3.** Factors that fall under the “mechanism of action” concept

Factors within the concept	Number of studies reporting factor	Barrier/facilitator/mixed	Summary of main findings for each factor as it applies to the audiology literature	Illustrative quotes from included studies
Knowledge	11	Facilitator	Sustained engagement with the DHI was facilitated when users perceived a meaningful acquisition of new knowledge [37, 47, 51, 55, 56, 66, 61, 70, 73, 76, 81]. Conversely, disengagement occurred when DHIs failed to contribute to users’ acquisition of new information [47]	“I’ve had a positive experience [of the program] because I think I now have some new information on how to deal with my hearing loss. (P1). While many participants revisited some previous theoretical and practical knowledge, many also learned some new things from participating in the program” [56].
Skill	7	Facilitator	DHIs facilitated engagement when users perceived a meaningful acquisition of hearing-related self-management skills [37, 66, 55, 59, 71, 76, 81]	“Participants noted how self-tuning facilitated core hearing loss self-management skills such as problem solving, coping mechanisms, and use of hearing aids. In doing so, participants commented that smartphone-connected hearing aids increased confidence in their hearing ability, cascading into other behaviors, such as increased participation” [59]
Motivations	7	Facilitator	Users were motivated to engage with the DHI when they perceived a sense of benefit in using the DHI [37, 47, 66, 59, 69, 71, 76]	“This was echoed by P3 who said: “I think it gave me a little bit more confidence knowing that…I’ve got that app I thought, ‘Oh, I can adjust it and get it to my level of hearing’.” This led to real-time adjustments using the app, with P1 noting: “Once I’d made the adjustment in wherever I was, I don’t think I hardly ever ignored a question. I could hear it all, maybe not- the only thing is, the last bit, but most of it, yes, almost anywhere.” P7 also noted the benefits of the app were extended to the workplace: “For me, in meetings, it was amazing, because I could actually hear what was being said. Normally, I couldn’t focus on it, but with a bit of adjustment, I could hear.”” [59]

#### Population

*N* = 28 studies reported on factors related to population (i.e. factors pertaining to the individual using a DHI). We observed that personal relevance, expectations, self-efficacy related to age and digital literacy and experience-of-wellbeing, mental health, language, physical limitations and age were identified under this concept (Table 4).

**Table 4.** Factors that fall under the “population” concept

Factors within the concept	Number of studies reporting factor	Barrier/Facilitator/Mixed	Summary of main findings for each factor as it applies to the audiology literature	Illustrative quotes from included studies
Personal relevance	19	Facilitator	Allowing users the opportunity to receive personally relevant content facilitated engagement across a wide range of DHIs [35, 37, 47, 51, 89, 53, 66, 56, 59, 62, 63, 71, 73, 76, 78, 80, 83, 90, 91]	“The short mRLOs enabled participants to locate personally relevant information with ease: I think the value is that you can just go to the subjects you want. For bits that you’re not interested in, well, what’s the point of going to that? What you want is information, and to be able to go straight to that bit of information, I think, is a valuable part of [m2Hear]. [M064] The succinct mRLOs also encouraged access and re-engagement, “They’re concise and logical, and that’s why I went back to them a couple of times” (M065). Furthermore, the mRLOs were a good use of time and effort” [76]:
Expectations	3	Barrier	Negative expectations about the privacy options and credibility of the DHI were barriers to engagement [66, 88, 86]	“Opinions among participants were divided with regard to the disclosure of personal information such as medical history and allergies in the app. Although one group found no problem in providing personal information, the other was uncertain. Some felt that it was acceptable to share their personal information on the app as long as it was only viewed by doctors and pharmacists. A similar concern was voiced when participants were asked if they preferred filling up forms through the app or in person. One participant said that he would prefer to fill it in person and pass it to the person at the counter himself, as he was afraid his personal data may be divulged to non-healthcare parties. Participants also highlighted data security concerns, similar with a survey of approximately 12,000 adult patients, where more than 50% had misgivings about using health IT due to concerns about data privacy” [86]
Experience of wellbeing	8	Facilitator	The negative psychosocial impacts associated with hearing loss and tinnitus (e.g. annoyance, communication breakdowns, distress), and dissatisfaction with current treatment options, facilitated the user’s initial decision to engage with DHIs [37, 66, 57, 71, 75, 76, 80, 87]	“Users with tinnitus accessed the intervention when they were either experiencing high levels of tinnitus distress or struggling to manage their tinnitus” [66].
Self-efficacy	6	Barrier	Participants negative perception of their age and digital literacy acted as barriers to engagement with DHIs [52, 59, 70, 71, 75, 76]	“Some participants commented that smartphone-connected hearing aids would be best utilized by a younger generation. P4 said: “I can understand that a younger generation, constantly used to being on their phone all the time, would find it absolutely brilliant, but for me I just thought, ‘Oh!’. Similarly, P7 stated: “One would hope that this would be available, if it’s going to be available on the NHS, for people of a much younger age group, and that’s who would be much more capable and wanting to use all of the things on it” [59].
Mental health	2	Facilitator	Inadequate coping with the psychosocial impacts of tinnitus facilitated initial decisions to engage with tinnitus DHIs [37, 66]	“Users with tinnitus accessed the intervention when they were either experiencing high levels of tinnitus distress or struggling to manage their tinnitus” [66].
Physical limitations	2	Barrier	Reduced agility and conditions such as Parkinson’s in older age contributed to older adults making more typing mistakes when using DHIs [89, 70]	“As older adults tend to be less agile with their hands (or fingers) than younger people, they made some typing errors despite understanding the target sound. We need to update the mobile program applying a pencil or pen instead of relying on the subject’s ability to type” [89].
Language	2	Barrier	Recognising that not all members of the Deaf community are native English speakers, facilitating engagement entails delivering content in sign language to ensure accessibility for individuals with diverse linguistic backgrounds [86, 80].	Six participants acknowledged that since not all Deaf individuals were literate, or could not fully understand the meaning of what they had read, the voice-to-text conversion may not be helpful. For that reason, some suggested providing sign language interpretation alongside the voice to-text conversion [86]
Literacy	2	Barrier	Members of the Deaf community may not be literate in reading English, serving as a potential barrier to engagement if the DHI is delivered in English [89, 80].	Writing is an ineffectual technique to deliver health information to Deaf patients because many Deaf people are less skilled in reading and writing. Heavy text content with jargon and complicated terminology will lose their attention [89]
Age	1	Barrier	Decreased agility in older age resulted in older adults making more typing mistakes when using an auditory training app. This limitation hindered their ability to effectively engage with the DHI [89]	“As older adults tend to be less agile with their hands (or fingers) than younger people, they made some typing errors despite understanding the target sound. We need to update the mobile program applying a pencil or pen instead of relying on the subject’s ability to type” [89].

#### Setting

*N* = 23 studies reported on factors related to setting (i.e. factors pertaining to the broader social context in which the user uses a DHI). Factors linked to engagement such as interruptions, healthcare systems location, time, and norms were discussed under this concept (Table 5).

**Table 5.** Factors that fall under the “setting” concept

Factors within the concept	Number of studies reporting factor	Barrier/Facilitator/Mixed	Summary of main findings for each factor as it applies to the audiology literature	Illustrative quotes from included studies
Interruptions	15	Barrier	Technical interruptions that disrupted app use and the perception of the app as interruptive to users’ everyday lives, served as barriers to engagement [92, 47, 51, 53, 56, 88, 58, 59, 69, 70, 77, 73, 78, 83, 90].	One participant was not able to connect the tablet computer with the Wi-Fi connection at home, and as a consequence, the MHA could not be launched [47]
Healthcare system	6	Mixed	Negative experiences with healthcare professionals were found to influence the initial decision to engage with some DHIs [86, 68, 80, 87]. Some users cited negative healthcare experiences as a motivating factor for adopting a tinnitus DHI [66].Conversely, if users perceived DHIs as unnecessary in their hearing healthcare journey, it served as a barrier to engagement [70].	“Some of these users were motivated by a perceived lack of support from health professionals, long waiting lists to see those professionals, and inadequacies of the health services in their country” [66].
Location	5	Mixed	Participants demonstrated engagement with the DHI when the location was conducive to use (e.g. a location where the DHI could be accessed or at home). Conversely, locations not conducive to DHI use (e.g. work meetings) acted as barriers to engagement [66, 59, 76, 93, 65]	“”[In] some situations, the use of a smartphone to control the hearing aid was acceptable, with P3 stating: “I felt quite at home getting my phone out. They didn’t know what I was dipping into or anything” [59].
Time	4	Barrier	Users were less likely to persist and engage with DHIs when they perceived the DHI as time-consuming [66, 88, 70, 73]	“‘[My] lifestyle does not promote or lend itself to periods of quiet reflection/relaxation.’ He therefore did not achieve any of the relaxation goals and reported not gaining any benefit from the intervention” [66]
Norms	1	Facilitator	The acceptability of using smartphones on various occasions facilitated the use and engagement of a self-fitting hearing aid app [59]	For example, P5 commented: “If it was in a meeting… that could be almost embarrassing, because you’re fiddling with your phone and, ‘Why are you on your phone?’ ‘Well, actually, I’m trying to listen to what you’re saying’.” Thus, perceived group or societal norms directly influenced whether alterations to smartphone-connected hearing aids were made or not [59]

## Discussion

The purpose of the review was to identify the different factors linked to engagement with DHIs in audiology, and their implications for future DHI development and research. A wide range of factors associated with engagement with DHIs were identified across the audiology literature. However, the included studies did not extensively study engagement nor primarily focus on engagement as compared to the evidence base for reviews examining engagement with DHIs in cognate fields like mental health [46, 94, 95], weight-loss management [96], cancer [97], and chronic disease management [45]. This observation aligns with the emergent nature of engagement as a concept in audiology, where in-depth exploration remains scarce in comparison to more established fields like mental health and chronic disease management.

### Concepts associated with engagement

#### Content

The content concept was one of the least frequently discussed concepts; this finding could be attributed to two possible reasons. First, DHI content, in itself, might not be important in facilitating engagement, rather, how content is delivered (e.g. personalised, easy-to-understand content) might be more important to engagement. This aligns with the broader literature on engagement with DHI, wherein discussions around influence of content on engagement is minimal [46, 94, 96]. However, it’s also important to consider that our analysis was grounded in a framework designed for behaviour change DHIs, which may have influenced our ability to capture content-related factors outside of behaviour change contexts. Therefore, future research should investigate whether content plays a more significant role in hearing health DHIs to better understand its relation to engagement.

#### Delivery

Delivery was the most frequently reported concept associated with engagement. This finding is consistent with patterns across reviews on DHI engagement in other fields wherein factors which fall under the delivery concept such as personalisation, guidance, and ease-of-use, were more frequently identified and discussed [46, 94, 96]. The frequent reporting of delivery factors does not indicate that delivery is the most important in promoting engagement with DHIs. Rather, their frequency can be attributed to the preponderance of studies that evaluate the DHI and features (i.e. studies evaluating whether content is appropriately delivered to users).

The factors related to the delivery concept highlighted that engagement can be facilitated by simplifying the DHI interface and the information provided by the DHI for users (e.g. by improving ease-of-use, guidance, personalisation and reducing complexity), consistent with previous literature [98–101]. Existing literature indicates that digital literacy, in itself, may not be a substantial barrier to DHI adoption; rather, it is the complexity of the interface that poses a challenge for users with lower levels of digital literacy [98, 100]. Simplifying the DHI can serve as a strategy to reduce the active effort and cognitive load required to engage with the DHI [102, 103]. This aligns with the Technology Acceptance Model where perceived ease of use (i.e. an individual’s belief that using a particular technology requires minimal effort) positively influences users’ decisions to adopt new technology [104]. Designing DHIs to be user-friendly and uncomplicated not only enhances their perceived ease of use, thereby promoting adoption [103], but it could potentially also increase user engagement.

#### Mechanisms of action

Mechanisms of action emerged as one of the least frequently discussed concept. This underreporting may be attributed to the fact that discussions on mechanisms of action were most frequently found in the limited number of studies that explored real-life user experiences with DHIs at various time points. Under the mechanisms of action concept, we identified that improving users’ knowledge and skills about hearing loss can facilitate engagement. These discussions highlight the role some aspects of empowerment play in facilitating engagement with DHIs. Empowerment in audiology is the process through which adults with hearing difficulties acquire and use knowledge and skills, increase participation, and the feeling of control over their hearing healthcare [105]. It is well established in audiology that DHIs empower users by enabling them to assert control over their hearing health [17, 19, 59, 106, 107]. We observed that users engaged with DHIs because users considered DHIs as a source of knowledge for hearing loss self-management [73, 76, 81], and users disengaged from DHIs if they felt that the DHI did not provide them with adequate information and knowledge [73, 76, 81]. This finding indicates a shift wherein users are no longer only receptively empowered by the DHI, but actively seek empowerment in engaging with DHIs. Notably, not meeting user expectations on empowerment could potentially impede engagement.

To the best of our knowledge, this is the first study highlighting a relationship between empowerment and DHI engagement. However, the current evidence is limited, and it is unclear what impact other dimensions of empowerment, such as participation and a sense of control might have on DHI engagement. The relationship between empowerment and engagement further raises questions about causality and mutual reinforcement, which warrants further research.

#### Population

The population concept revealed that engagement with DHIs is facilitated when DHI use aligns with users’ physical, psychosocial, cognitive, and hearing-related needs and expectations. We observed that adults with hearing difficulties are more likely to engage with tinnitus DHIs if they are more distressed or psychosocially impacted by their tinnitus [37, 66, 86, 80]. Similar patterns emerge in studies on mental health DHIs, where users with more severe mental health symptoms were more willing to engage with mental health DHIs [107–109]. However, other studies suggested that severe depression scores act as barriers to engagement with mental health DHIs [110–112]. The contradicting results could be explained by the fact that it is not necessarily the severity of the mental health symptom associated with the health condition that acts as a barrier, rather, low mood and other depressive symptoms might impede an individual’s motivation to engage with a DHI. Interestingly, a clinician-supported DHI for tinnitus management revealed that high depression scores relate poorly with DHI engagement [112]. While poorly coping with tinnitus can facilitate engagement with tinnitus DHIs, low mood and depression consequent to tinnitus might act as barriers to engagement. Overall, the relationship between symptom severity, mental health and DHI engagement appears interrelated and complex, warranting further research.

Further, it is unclear whether an increase in symptom severity only facilitates the user’s initial decision to engage or if it facilitates sustained engagement with a DHI. Considering broader literature, symptom severity influenced the initial decision to engage with a DHI for post-traumatic stress disorder management, while sustained engagement was associated with perceived improvements in symptoms among other factors [113]. It is plausible that this might also apply to DHIs in audiology wherein perceived severity of hearing-related symptoms might contribute to initial engagement, while sustained engagement might be facilitated by user’s perceived sense of benefit. This notion gains further support from the evidence on hearing aids, which suggests that perceived severity of hearing difficulties is a robust indicator of hearing aid uptake [114–118], while perceived hearing aid benefit and satisfaction with hearing aids are strong predictors of consistent hearing aid use [119, 120]. However, the relationship between symptom severity and sustained engagement with DHIs in audiology warrants further research before robust conclusions can be reached.

Further, we observed that negative expectations surrounding DHI use originate from preconceptions about aging and poor digital literacy, rather than actual user experience with technology, consistent with another study [121]. This emphasises the importance of enhancing users perceived self-efficacy (i.e. their belief in their ability to use DHIs) to promote engagement. According to social cognitive theory, an individual’s self-efficacy can be improved through mastery experience (individuals persevering through obstacles), social modelling (seeing people like oneself succeed by perseverant effect), and social persuasion (encouragement to believe in oneself) [122]. When developing or recommending DHIs to adults with hearing loss, incorporating strategies that actively enhance self-efficacy could be beneficial in improving user engagement with DHIs.

#### Setting

The setting concept emphasised that DHIs should seamlessly integrate into users’ lifestyle and should not be perceived as disruptive to facilitate engagement. If DHI use disrupted an individual’s everyday routine, participants seldom engaged with the DHI [66]. In line with this, a systematic review on factors influencing engagement with mental health DHIs identified lack of time, non-conducive settings, and inability to establish a routine that worked for participants as barriers to engagement [94]. The findings support a person-based approach to DHI development, which emphasises the importance of considering users’ wants and needs in the development process to minimise disruptions to users’ daily lives and maximise effectiveness in real-world settings [123].

## Limitations

This review aimed to examine both qualitative and quantitative literature to examine the factors linked to engagement with DHIs designed for adults with hearing loss and tinnitus. However, because of the heterogeneity of the included study designs and the lack of studies specifically exploring engagement, we were unable to conduct a meta-analysis. Further, the methodological assessment in the review examines the quality of research methods and does not examine the quality of evidence on engagement. Although an evidence-based framework was used to guide data extraction and improve the validity of the systematic review, our findings are based on studies that did not explore engagement as the primary outcome. As such, inferences had to be made on secondary outcomes and discussions from available literature. It is thus difficult to assess the methodological quality of discussions on engagement. To allow for more robust discussions, conclusions and causal inferences on engagement, studies that primarily explore how adults with HL engage with DHIs are necessary in audiology.

## Conclusion

This is the first systematic review in audiology to synthesise both quantitative and qualitative evidence and summarise the current understanding of engagement with DHIs in audiology. The review identified various factors associated with engagement in hearing-related DHIs, emphasising the importance of user-centred constructs such as improving accessibility, empowering users, and addressing their needs, lifestyle, and expectations. The findings underscore the importance of adopting a user-centred approach to DHI development and evaluation in audiology. Due to the limited number of studies focusing on engagement as the primary outcome, we based our inferences on secondary outcomes and discussions from the available literature. While our review consolidates existing knowledge on engagement, it also highlights the need for further research to primarily explore engagement to strengthen the current evidence and help make causal inferences about factors influencing engagement in audiology.

## Supplementary Material

ibaf028_suppl_Supplementary_Materials_1

ibaf028_suppl_Supplementary_Materials_2

ibaf028_suppl_Supplementary_Materials_3

ibaf028_suppl_Supplementary_Materials_4

ibaf028_suppl_Supplementary_Materials_5
