# Supplementary material for: Understanding engagement with digital health interventions designed for adults with hearing loss and tinnitus: a mixed-method systematic review
Source: Transl Behav Med. 2025 Jun 30;15(1):ibaf028. doi: 10.1093/tbm/ibaf028 (PMC12207976; doi:10.1093/tbm/ibaf028)
Supplement: ibaf028_suppl_Supplementary_Materials_2 [file ibaf028_suppl_supplementary_materials_2.docx]

**Ovid MEDLINE(R) ALL**

1 ("self-fitting hearing aid*" or "self-fitting hearing aid").mp. [mp=title, book title, abstract, original title, name of substance word, subject heading word, floating sub-heading word, keyword heading word, organism supplementary concept word, protocol supplementary concept word, rare disease supplementary concept word, unique identifier, synonyms, population supplementary concept word, anatomy supplementary concept word] 17

2 exp Audiology/ 2371

3 exp Hearing Loss/ 78843

4 "hearing aid user".mp. 68

5 "cochlear implant user".mp. 63

6 exp Deafness/ or deaf.mp. 36160

7 hearing impairment.mp. 13245

8 hearing care.mp. 235

9 hearing related.mp. 439

10 exp Presbycusis/ 1677

11 hearing health.mp. 688

12 exp "Correction of Hearing Impairment"/ or aural rehab*.mp. or hearing rehab*.mp. 6858

13 (internet intervention or internet based intervention).mp. 2221

14 Health Promotion/ or mobile intervention.mp. 82230

15 exp Digital Technology/ 796

16 exp Mobile Applications/ 11954

17 "Delivery of Health Care"/ 119956

18 ("e health" or "e-health" or "ehealth").mp. 11583

19 ("mhealth" or "m-health" or "m health").mp. 10824

20 exp Smartphone/ 9512

21 web based intervention.mp. 1194

22 1 or 2 or 3 or 4 or 5 or 6 or 7 or 8 or 9 or 10 or 11 or 12 92877

23 13 or 14 or 15 or 16 or 17 or 18 or 19 or 20 or 21 237466

24 22 and 23

**585**

**CINAHL**

"self-fitting hearing aid*" OR "self fitting hearing aid*" OR ((audiologi* OR audiology OR "hearing loss" OR "hearing aid user" OR "cochlear implant user" OR "deaf" OR deafness OR hearing impairment OR hearing care OR "hearing related" OR presbycusis OR hearing health OR "hearing rehab*") AND ("web-based intervention" OR "internet intervention" OR "internet based intervention" OR "mobile intervention" OR "digital technology" OR "mobile application" OR "mobile app" OR "e-health" OR "ehealth" OR "m-health" OR "mhealth" OR "smartphone")) NOT (children OR surgery OR pediatric OR animal OR gene OR diabetes)

**257**

**PUBMED**

"self-fitting hearing aid*" OR "self fitting hearing aid*" OR ((audiologi* OR audiology OR "hearing loss" OR "hearing aid user" OR "cochlear implant user" OR "deaf" OR deafness OR hearing impairment OR hearing care OR "hearing related" OR presbycusis OR hearing health OR "hearing rehab*") AND ("web-based intervention" OR "internet intervention" OR "internet based intervention" OR "mobile intervention" OR "digital technology" OR "mobile application" OR "mobile app" OR "e-health" OR "ehealth" OR "m-health" OR "mhealth" OR "smartphone")) NOT (children OR surgery OR pediatric OR animal OR gene OR diabetes)

**347**

**APAPsycinfo**

APA PsycInfo <1806 to December Week 1 2023>

1 (self-fitting hearing aid or self fitting hearing aid).mp. 2

2 exp Audiology/ 1088

3 exp Sensorineural Hearing Loss/ or exp Hearing Loss/ or exp Deafness/ 17103

4 hearing aid user.mp. 34

5 cochlear implant user.mp. 11

6 hearing impairment.mp. 3739

7 hearing care.mp. 74

8 presbycusis.mp. 485

9 hearing health.mp. 249

10 (aural rehabilitation or hearing rehabilitation).mp. 326

11 web based intervention.mp. 652

12 exp Digital Interventions/ 1682

13 (internet intervention or internet based intervention).mp. 849

14 mobile intervention.mp. or exp Health Promotion/ 29192

15 digital technology.mp. 3811

16 exp Mobile Applications/ 2748

17 (ehealth or e-health or e health).mp. 3072

18 (mhealth or m-health or m health).mp. 2312

19 Smartphones/ 3385

20 1 or 2 or 3 or 4 or 5 or 6 or 7 or 8 or 9 or 10 20933

21 11 or 12 or 13 or 14 or 15 or 16 or 17 or 18 or 19 44354

22 20 and 21 **105**

**Global Health**

Global Health <1910 to 2023 Week 50>

1 Audiology.mp. 206

2 Hearing loss.mp. 4666

3 deaf*.mp. 3375

4 Hearing impair*.mp. 5059

5 hearing care.mp. 37

6 hearing health.mp. 115

7 (aural rehab* or hearing rehab*).mp. 39

8 hearing health.mp. 115

9 presbycusis.mp. 69

10 presbycusis.mp. 69

11 (web based intervention or internet intervention or internet based intervention).mp. 297

12 Health promotion.mp. [mp=abstract, title, original title, heading words, cabicodes words] 39336

13 mobile intervention.mp. 26

14 digital technology.mp. 2361

15 mobile application*.mp. 1385

16 delivery of healthcare.mp. 229

17 (e health or e-health or ehealth).mp. 1410

18 (mhealth or m-health or m health).mp. 1426

19 smartphone.mp. 2764

20 1 or 2 or 3 or 4 or 5 or 6 or 7 or 8 or 9 8371

21 10 or 11 or 12 or 13 or 14 or 15 or 16 or 17 or 18 or 19 47406

22 20 and 21 **192**

**Embase**

1 (self-fitting hearing aid* or self fitting hearing aid*).mp. 18

2 exp hearing/ or audiology.mp. or exp hearing impairment/ or exp audiology/ 177599

3 hearing loss.mp. 83398

4 hearing aid user.mp. 78

5 cochlear implant user.mp. 69

6 Deafness.mp. 59465

7 deaf.mp. 13756

8 hearing impairment.mp. 78927

9 hearing care.mp. 282

10 hearing related.mp. 473

11 presbycusis.mp. 1474

12 (aural rehab* or correction of hearing impairment).mp. 442

13 exp web-based intervention/ 2984

14 internet intervention.mp. 545

15 internet based intervention.mp. or exp web-based intervention/ 3542

16 exp health promotion/ 117891

17 mobile intervention.mp. 189

18 exp digital technology/ 4830

19 exp mobile application/ 26682

20 (ehealth or e-health or e health).mp. 12878

21 (mhealth or m-health or m health).mp. 10165

22 hearing health.mp. 790

23 1 or 2 or 3 or 4 or 5 or 6 or 7 or 8 or 9 or 10 or 11 or 12 or 22 210742

24 14 or 15 or 16 or 17 or 18 or 19 or 20 or 21 or 13 166871

25 23 and 24 726

26 limit 25 to (adult <18 to 64 years> or aged <65+ years>) **326**

**EBM Reviews - Cochrane Central Register of Controlled Trials**

1 exp Hearing Loss/ 1620

2 hearing aid user.mp. 14

3 exp Audiology/ 42

4 self-fitting hearing aid.mp. 3

5 cochlear implant user.mp. 2

6 exp Hearing Loss/ 1620

7 exp "Correction of Hearing Impairment"/ 154

8 (aural rehabilitation or hearing rehabilitation).mp. 80

9 exp Presbycusis/ 40

10 hearing health.mp. 47

11 exp Internet-Based Interventions/ 565

12 digital intervention.mp. 413

13 mobile intervention.mp. 149

14 exp Health Promotion/ 8260

15 exp Digital Technology/ 28

16 (e health or e-health or ehealth).mp. 1697

17 (mhealth or m-health or m health).mp. 2499

18 exp Smartphone/ 1046

19 1 or 2 or 3 or 4 or 5 or 6 or 7 or 8 or 9 or 10 1794

20 11 or 12 or 13 or 14 or 15 or 16 or 17 or 18 14146

21 19 and 20 **15**
