# Supplementary figures and images for: Understanding engagement with digital health interventions designed for adults with hearing loss and tinnitus: a mixed-method systematic review
Source: Transl Behav Med. 2025 Jun 30;15(1):ibaf028. doi: 10.1093/tbm/ibaf028 (PMC12207976; doi:10.1093/tbm/ibaf028)

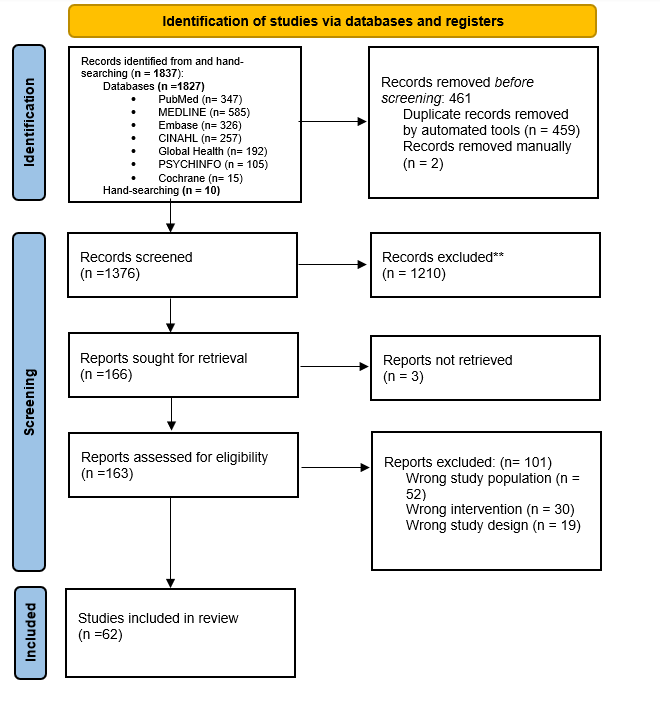

Supplement: ibaf028_suppl_Supplementary_Materials_3 [file ibaf028_suppl_supplementary_materials_3.docx]
